# Identification of circRNA-miRNA-mRNA networks to explore the molecular mechanism and immune regulation of postoperative neurocognitive disorder

**DOI:** 10.18632/aging.204348

**Published:** 2022-10-21

**Authors:** Ning Bao, Jiping Liu, Zhe Peng, Rong Zhang, Rufei Ni, Runzuan Li, Jian Wu, Zhenhua Liu, Botao Pan

**Affiliations:** 1 Department of Anesthesiology, Affiliated Foshan Maternity and Child Healthcare Hospital, Southern Medical University, Foshan, Guangdong, China; 2 Department of Anesthesiology, Shenyang Women’s and Children’s Hospital, Shenyang, Liaoning, China

**Keywords:** postoperative neurocognitive disorder (PND), bioinformatics, transcriptome, competitive endogenous RNA network, immune cell infiltration

## Abstract

Postoperative neurocognitive disorder (PND) is a common complication in older patients. However, its pathogenesis has still remained elusive. Recent studies have shown that circular RNA (circRNA) plays an important role in the development of neurodegenerative diseases, such as PND after surgery. CircRNA, as a competitive endogenous RNA (ceRNA), mainly acts as a molecular sponge for miRNA to “adsorb” microRNA (miRNA) and to reduce the inhibitory effects of miRNAs on target mRNA. The sequencing data of circRNA were obtained from the Gene Expression Omnibus (GEO) database.

By bioinformatic methods, circAtlas, miRDB, miRTarBase and miRwalk databases were applied to construct circRNA-miRNA-mRNA networks and screen differentially expressed mRNAs. To improve the accuracy of the data, we randomly divided aging mice into control (non-PND group) and PND groups, and used high-throughput sequencing to analyze their brain hippocampal tissue for analysis. Three key genes were cross-detected in the data of both groups, which were Unc13c, Tbx20 and St8sia2 (as hub genes), providing new targets for PND treatment. According to the results of the Gene Ontology (GO) and Kyoto Encyclopedia of Genes and Genomes (KEGG) pathway enrichment analyses, immune cell infiltration analysis, gene set enrichment analysis (GSEA), Connectivity Map (CMap) analysis, quantitative real-time polymerase chain reaction (qRT-PCR), the genes that were not related to the central nervous system were removed, and finally, mmu_circ_0000331/miR-1224-3p/Unc13c and mmu_circ_0000406/miR-24-3p/St8sia2 ceRNA networks were identified. In addition, the CMap method was used to select the top 4 active compounds with the largest negative correlation absolute values, including cimaterol, Rucaparib, FG-7142, and Hydrocortisone.

## INTRODUCTION

Postoperative neurocognitive disorder (PND) is a series of clinical manifestations characterized by inattention, decreased language comprehension, cognitive decline, and difficulty in returning to preoperative life after surgery [1]. PND includes acute delirium and more lasting postoperative neurocognitive impairment [2], mainly occurring in old patients [3], and it has become a major public health concern. International studies on postoperative cognitive function estimated that the one-year mortality rate of PND patients within three months after surgery was almost twice that of patients without PND [4]. The risk of PND in old patients was reported to be 25–40% [5]. The global prevalence of dementia was 46.8 million in 2015, and it is expected to increase to 131.5 million by 2050. The global cost of dementia was estimated to be $818 billion in 2015 [6]. PND leads to delayed recovery, prolonged hospitalization, increased medical costs, enhanced complications, and even loss of self-care ability, resulting in a series of medical, social, and economic problems [7]. Several studies have explored the possible mechanism of PND, and neuroinflammation [8], neuronal apoptosis [9], autophagy disorders [10], and synaptic plasticity [11] have been reported. At present, the pathogenesis of PND is not clear, and there is no effective treatment and specific marker for PND.

With the rapid development of molecular biology and transcriptology, the biological functions and regulatory mechanisms of non-coding RNA can explain the occurrence and development of several complex diseases. Circular RNA (circRNA) is a newly discovered class of non-coding RNA with a length of about 100 nucleotides. The circRNA molecule has no PolyA tail with 5′ and 3′ ends, and it has a closed ring structure with a high stability. The roles of circRNAs in various diseases have been widely studied due to their unique molecular mechanism and molecular function, and have become a research hotspot. Their main function is to act as a molecular sponge for microRNAs (miRNAs) to “adsorb” miRNAs and to reduce the inhibition of miRNAs on target mRNAs. The mechanism of competitive endogenous RNAs (ceRNAs) in the occurrence and development of diverse diseases has been most thoroughly studied. Studies have reported that ceRNA network could have a great potential in the treatment of the central nervous system (CNS) diseases [12]. Recent studies have shown that circRNA_22058 and circRNA_44122/EGFR ceRNA network and circRNA_22673/Prkacb ceRNA could be the mechanism of PND [13]. However, the role of circRNA in elderly patients with PND has still remained elusive.

**Figure 1:**
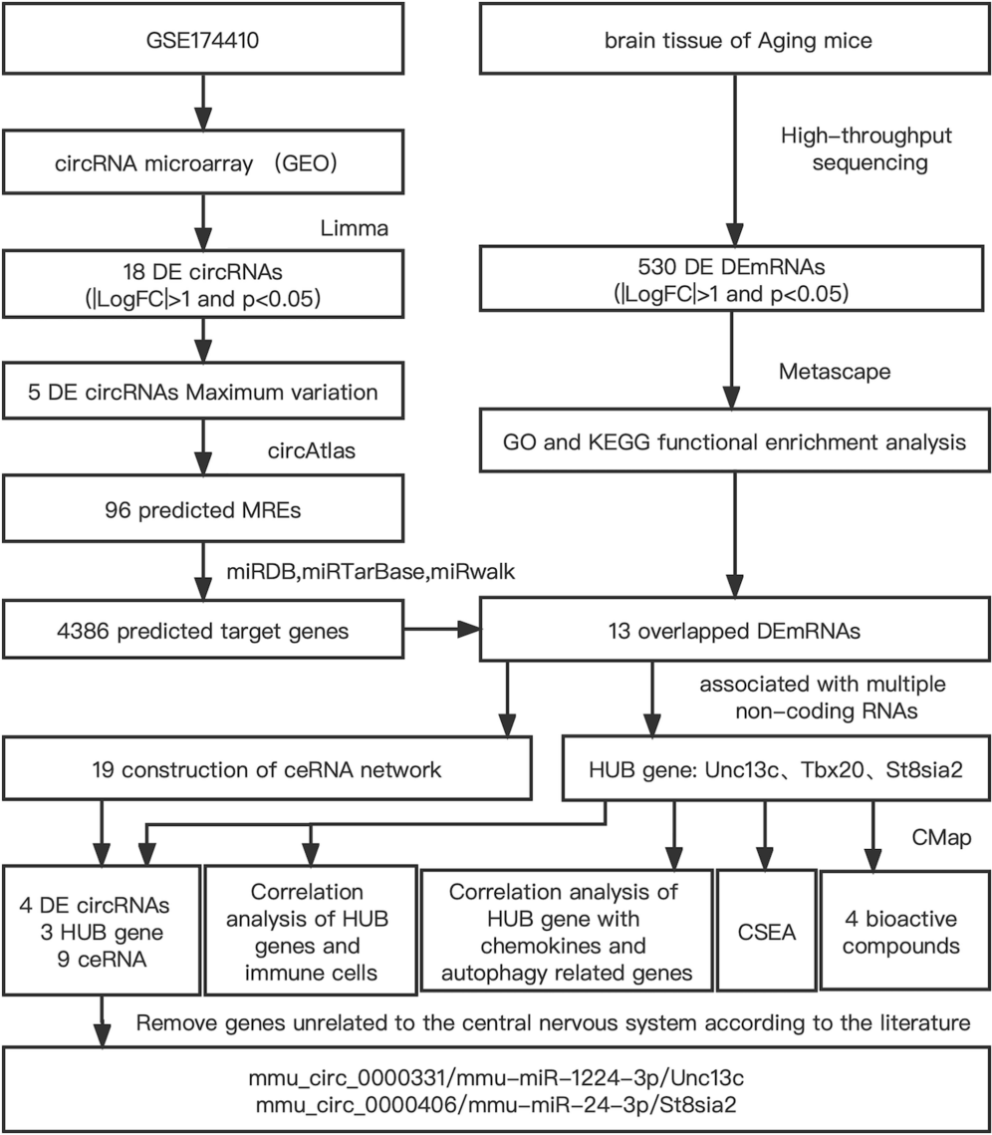
Flowchart of the comprehensive analysis process and all methods utilized in present study.

Bioinformatics research has revealed the molecular targets of PND mechanism in a systematic, accurate, and effective manner, and clarified the theoretical basis of PND occurrence. In the present study, prefrontal cortex data of PND in aged mice obtained from GEO database were screened, and a circRNA-miRNA-mRNA network was constructed by bioinformatics method to identify differential mRNAs. To further improve the accuracy of data, the hippocampus of splenectomized aged mice were sent to Novogene Co., Ltd. (Beijing, China) for high-throughput sequencing of mRNA. The two sets of data were intersected to detect hub genes. Surgery and trauma can trigger inflammatory responses characterized by both pro-inflammatory and anti-inflammatory cytokines released by the immune system [14]. A large number of studies have shown that immune cells trigger neuroinflammatory responses, leading to postoperative cognitive dysfunction [15]. Immune regulation has become a hotspot in the study of PND mechanism. The present research aimed to identify immune-related genes. The flowchart of this study is shown in Figure 1.

## METHODS

### Animals

Healthy male C57BL/6 mice (age, 18-month-old; body weight, 25–30 g) were purchased from Beijing Weitong Lihua Co., Ltd. (Beijing, China). All animals were acclimated for one week before the experiment. All animal experiments were performed in accordance with the Guide for Care and Use of Laboratory Animals, and approved by the Animal Care and Use Committee of the Southern Medical University (Guangzhou, China). The aging mice were divided into two groups: control group (*n* = 11) and surgery group (*n* = 11). Mice in the surgery group received splenectomy under general anesthesia. Among them, 16 mice underwent Morris water maze (MWM) test to determine the cognitive dysfunction in aging mice after surgery, and the hippocampus of the remaining 6 mice were taken out for transcriptome detection.

### Establishment of animal models

Splenectomy was performed under inhalation anesthesia (maintained by a mixture of 2.0–2.5% isoflurane and oxygen). The anal temperature was maintained between 38.8–39.8°C. After undergoing anesthesia, the left subcostal incision was made. The spleen was separated and exposed, the spleen arteries and veins in the splenic pedicle region were firmly ligated, and the spleen was removed. After confirming no active bleeding, the abdomen was closed layer-by-layer with silk thread. Mice in the control group were not anesthetized or underwent surgery. Splenectomy leads to reversible postoperative learning and memory dysfunction and subsequently induces Postoperative cognitive dysfunction (POCD) [16], and is often used as a model for establishing POCD [17].

### MWM test

MWM test was employed to assess abilities of mice in learning, memory, and spatial cognition. A pool with a diameter of 120 cm and a depth of 50 cm was divided into four quadrants, and a platform with a diameter of 12 cm was set 1 cm underwater in the target quadrant. Experimental mice first received positioning navigation training for 5 days. Mice were placed on the platform for 30 s, and were put into the pool from four different quadrants (N, W, S, and E). Observe and record the distance, time and speed of mice reaching the platform for the first time. If mice could not find the platform within 60 s, they were guided to the platform for 15 s. The interval between the two training sessions was at least 30 s. The time of reaching the platform for the first time, the number of times that the distance and speed could cross the platform quadrant, and the number of times that they crossed the platform quadrant were recorded. The experiment was lasted for at least 30 s. The time to reach the platform for the first time, the distance and speed to cross the platform, and the number of times to cross the platform quadrant were recorded. On day 6 and postoperative days 3, 7, and 14, the platform was removed and the MWM assay was performed to record the distance, speed, and time spent across the platform for the first time and the percentage of time spent across the target quadrant. Finally, the time point of the most severe PND in the aging mice was selected as the time point for taking the materials. The test performers and data analyzers were blinded to the mice groups.

### Sample collection and sequencing

Under isoflurane deep anesthesia, 6 mice were killed by decapital method, including 3 mice in control group and 3 mice in surgery group. The skull was separated by non-sharp method, the hippocampus were placed on ice bags, and then, the hippocampus were frozen in liquid nitrogen and stored in a −80°C refrigerator. The hippocampus were randomly selected for subsequent sequencing analysis. Total RNA was extracted from mouse hippocampus using TRIzol^®^ reagent (Magen, Guangzhou, China). Nanodrop ND-2000 (Thermo Fisher Scientific, Waltham, MA, USA) was used to detect the A260/A280 absorbance ratio of RNA samples. An Agilent 4150 Bioanalyzer (Agilent Technologies, CA, USA) was used to measure RIN values of RNA. A PE library was prepared according to ABclonal mrna-SEQ Lib Prep Kit (ABclonal, China) specification. Sequencing was performed using the Illumina Novaseq 6000/MGISEQ-T7 sequencing platform (Illumina Inc., Chicago, IL, USA).

### Data collection and identification of DEcircRNAs and DEmRNAs

In the present study, GSE174410 dataset was downloaded from the GEO database (http://www.ncbi.nlm.nih.gov/geo), as well as annotation platforms for GPL21273, including 3 normal tissue samples and 3 post-operative neurocognitive impairment tissue samples, accounting for a total of 6 samples. In the present experiment, high-throughput sequencing was performed on the mRNA of hippocampus of aging mice, including 6 samples from control group (*n* = 3) and PND group (*n* = 3). The “Limma” package was used to standardize the data of the two groups, and the circRNA/mRNA was significantly differentially expressed in the control group and the PND group. A gene with |LogFC|>1 and *P* < 0.05 was considered to be significantly differentially expressed, and it was displayed through a volcano plot.

### Biological functional analysis

In order to obtain the biological functions and signaling pathways involved in the occurrence and development of PND, the Metascape database (http://www.metascape.org) was used to enrich and analyze 530 mRNAs with differences in the experimental data for annotation and visualization. Specific genes were analyzed by the Gene Ontology (GO) and the Kyoto Encyclopedia of Genes and Genomes (KEGG) pathway enrichment analyses. Min overlap ≥3 and *P* ≤ 0.05 were considered statistically significant.

### Construction of the circRNA-miRNA-mRNA network

We used circAtlas database to predict circRNA-miRNA interaction pairs. In addition, the interaction between miRNA and mRNA was predicted by combining data collected from miRDB, miRTarBase, and miRwalk databases. Targeted mRNAs identified by more than two databases were selected for further analysis. Then, the circRNA-miRNA-mRNA network was established by combining circRNA-miRNA interaction and mRNA-miRNA interaction, and Cytoscape software was used for visualization of biological networks.

### Immune infiltration analysis

The CIBERSORT algorithm was used to analyze RNA-seq data of mice in the control and PND groups to infer the relative proportions of 25 immune infiltrating cells. The “Pheatmap” package was used to draw the heatmap of immune cell infiltration to explore the distribution of immune cells. The “Corrplot” package was utilized to analyze the interaction between immune cells and to further analyze the influence of the interaction between immune cells. The “Vioplot” package was employed to plot the relative content of immune cells. *P* < 0.05 was considered statistically significant. Then, the Spearman correlation analysis was conducted between HUB gene expression level and immune cell content, and “GGploT2” package was used for visualization. Chemokines refer to small cytokines or signal proteins that have the function of making immune cells targeted to chemotaxis, which can control the migration and residence of all immune cells. Autophagy, as a defense mechanism of the collective, in the body’s immunity, inflammation, neural degeneration disease, aging, etc., has shown a very important role in the pathogenesis, and it is closely associated with the immune cells. We, in the present study, further discussed the hub genes and chemotactic factors, as well as the relationship between the autophagy using the Pearson correlation analysis.

### Gene set enrichment analysis (GSEA) of hub genes

Unc13c, Tbx20, and St8sia2 mRNA were analyzed by the GSEA. GSEA analyzes the expressions of a group of functionally-related genes based on gene expression profile data. The principle of GSEA is to search for sets of genes that are significantly over-represented in a given list of genes. According to the number of genes contained in the gene set and the expressions of genes, the normalized enrichment score (NES), false discovery rate (FDR), and adjusted *P* value of the signaling pathway were calculated. Genes with *P* < 0.05 were considered significantly enriched after 1000 permutations.

### Identification of co-expressed genes (Unc13c, Tbx20, and St8sia2)

The PPI networks of Unc13c, Tbx20, and St8sia2 were constructed and visualized separately based on STRING database. The correlation analysis was performed between hub genes and their co-expressed gene expression levels.

### RNA extraction and quantitative real-time polymerase chain reaction (qRT-PCR)

RNA was extracted and validated from the cerebral cortex of 5 control and 5 PND aging mice using qRT-PCR. Total RNA was extracted using TRIzol reagent (Cat. No. 1596–026; Thermo Fisher Scientific). The reverse transcription reaction system was prepared according to the instructions of Bestar qPCR RT Kit. The total system was 20 μL, and the first strand of cDNA was synthesized. Follow the instructions presented for DBI Bestar SybrGreen qPCR masterMix reaction kit, annealing was carried out at 94°C for 2 min, at 94°C for 15 s, and at 60°C for 15 s, at 72°C for 15 s, with 40 cycles. GAPDH was used as an internal reference gene. A QuantStudio3 fluorimeter (Thermo Fisher Scientific) was used for fluorescence quantitative analysis. Relative gene expression was calculated by the 2^−ΔΔCT^ method. The primers are shown in Table 1.

**Table 1:** Primer information.

**Target name**	**Primer**
Unc13c	F: AGTTACCGAGTTGCTATCGCC
	R: GCTGCTCCTTAGCTCATTGAA
St8sia2	F: TCGCTGACAGAAGTAATGAAAGC
	R: TCAGAGAGAGCGTCTGGTTGT
Tbx20	F: AAACCCCTGGAACAATTTGTGG
	R: CAGGCGATTTTAGCCATCTCTT
mmu_circ_0000331	F: TGTGCCCCTTGTGTTCTCAG
	R: AGCCAGTTTGAACCGGATGT
mmu_circ_0000400	F: CTCAGCTCTCACTGCACCAA
	R: TCACCAGAATCCCACGCTTC
mmu_circ_0000406	F: GGAGAACCTGCGAAAGAGGT
	R: TTGATGGGATCCAGTGTGGC
mmu_circ_0000798	F: GAGTTGTGTGGCCTCTCCTC
	R: TTCTGCTTCGTGCCATCCAT
GAPDH	F: AGGTCGGTGTGAACGGATTTG
	R: GGGGTCGTTGATGGCAACA

### Connectivity Map (CMap) analysis

CMap is a database of expression profiles based on the expression of intervening genes developed by the Broad Institute. Lamp proposed that it could be used to discover the association between drugs, genes and diseases, and constructed the CMap database when the gene changes were obtained after cell disturbance [18]. CMap was used to compare the data list of differentially expressed genes measured by our research group with the database reference data set. Finally, the database will obtain a correlation score (−100–100) according to the enrichment of differentially expressed genes in the reference gene expression spectrum. The positive value indicated that the up-regulated and down-regulated differential expressions were similar, while the negative value indicated that the up-regulated and down-regulated differential expressions were opposite, and the reference gene expression was sequenced according to the value.

### Statistical analysis

All data were analyzed using Graphpad Prism 8 software, and the measures were expressed according to the mean ± SD. Data were compared between groups using analysis of variance (Two-way ANOVA) *P*-value < 0.05 was considered statistically significant with great importance.

### Availability of data and materials

Part of datasets generated during and/or analyzed during the current study are available in the Gene Expression Omnibus (GEO) datasets (http://www.ncbi.nlm.nih.gov/geo/) and the other part of the data came from the high-sequencing results of our experiment.

## RESULTS

### Behavioral test results

**Figure 2:**
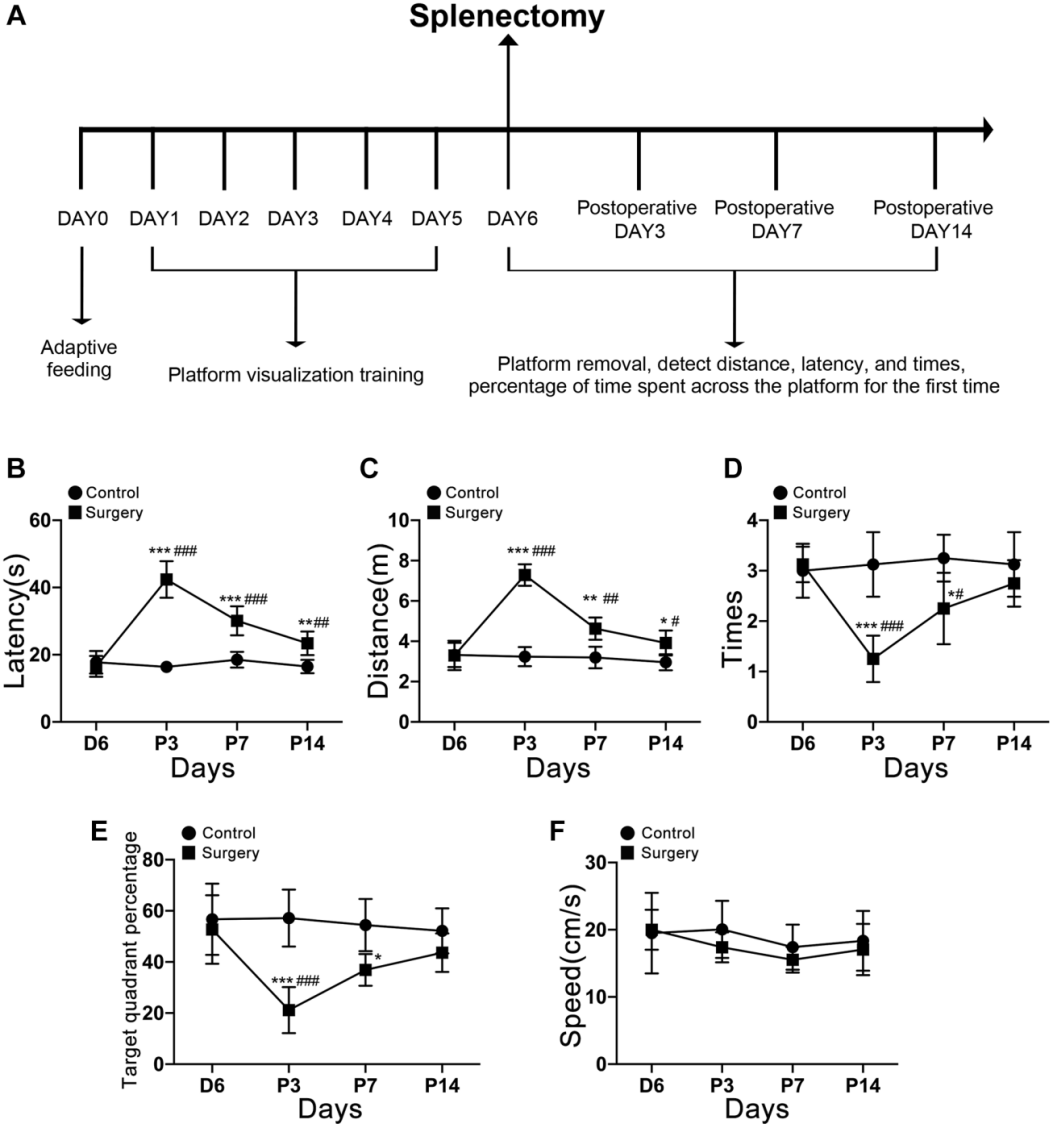
**Timeline diagram of the Morris Behavior Test.** (**A**) Morris water maze testing of aging mice 3, 7, and 14 days after splenectomy. (**B**) The time that the aging mice reach the platform for the first time. (**C**) The distance taken by the aging mice to reach the platform for the first time. (**D**) The number of times the platform was crossed in 60 s. (**E**) The percentage of the total time for the aging mice to cross the target quadrant. (**F**) There was no significant difference in swimming speed at each time point (*P* > 0.05). The data are presented as mean ± SEM. ^*^P < 0.05 indicated that there was a significant difference between the two groups at corresponding time points. ^#^*P* < 0.05 indicated that compared with C6, P3, P7, and P14 significantly changed. Control c. Surgery: Surgery group. C6: the incubation period of positioning voyage is day 6. P3, P7, P14: postoperative days 3, 7, and 14.

During the 6 days of preoperative training, all experimental mice showed a gradual downward luminal trend in the time and distance to find the platform, indicating that the experimental mice were able to improve their spatial learning memory ability by constantly repeating finding the platform. The aging mice showed a significantly impaired memory on postoperative day 3, as the time and distance to find the platform (Figure 2B, 2C) both significantly increased; the number of times to pass the platform within 60 s and the percentage of target quadrants (Figure 2D, 2E) both significantly decreased, while the average speed did not significantly differ (Figure 2F), and basically recovered on postoperative day 14. Comparing postoperative day 3 with preoperative training day 6, postoperative day 7, and postoperative day 14, postoperative day 3 was the day with the worst memory ability and the most obvious cognitive impairment, as evidenced by a significant increase in the time and distance to find the platform for the first time and a significant decrease in the number of times to pass the platform within 60 s and the percentage of target quadrants. Therefore, we selected the hippocampus of aging mice for transcriptomic assay on the 3rd postoperative day when memory impairment was most severe. Timeline diagram of the Morris Behavior Test is shown in Figure 2A.

### Differential expression of circRNAs and mRNAs

**Figure 3:**
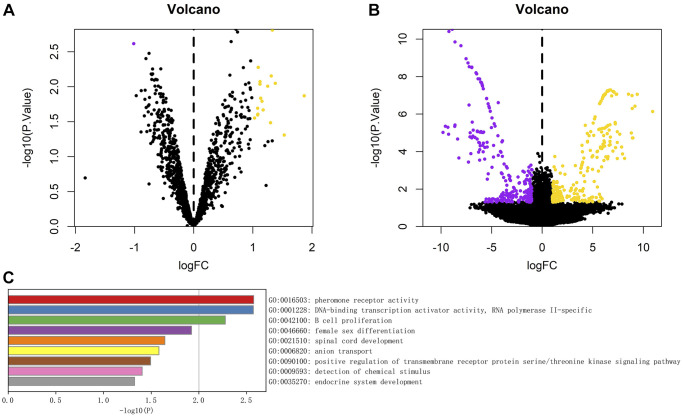
(**A**) Volcanic diagram of differential analysis of circRNA; (**B**) Volcanic map of mRNA differences detected in the experiment. The yellow dot represents the up-regulated genes, the purple dot represents the down-regulated genes, the abscissa is the log2 change multiple, and the ordinate is *P*-value. (**C**) Analysis of enrichment function of 530 differentially expressed genes, The length of the column represents the size of *P*-value.

A total of 18 differentially expressed circRNAs, including 17 up-regulated circRNAs and 1 down-regulated circRNA, were identified from the circRNA transcriptome data of GSE174410 downloaded from the GEO database (Figure 3A). Compared with the control group, the difference in circRNA expression in the surgical group was statistically significant. A total of 530 differentially expressed mRNAs, including 266 up-regulated mRNAs and 264 down-regulated mRNAs, were screened, which are represented as volcano plots (Figure 3B).

### The GO and KEGG pathway enrichment analyses

We further conducted the GO and KEGG pathway enrichment analyses of 530 differentially expressed mRNAs through the Metascape database, and the results showed that, the main gene enrichment pathways were pheromone receptor activity, DNA-binding transcription factor activity, RNA polymerase II-specific and B cell proliferation, female sex differentiation, spinal cord development, anion transport, positive regulation of transmembrane receptor serine/threonine kinase signaling pathway, detection of chemical stimulus, and endocrine system development (Figure 3C).

### Establishment of the circRNA-miRNA-mRNA network

**Figure 4:**
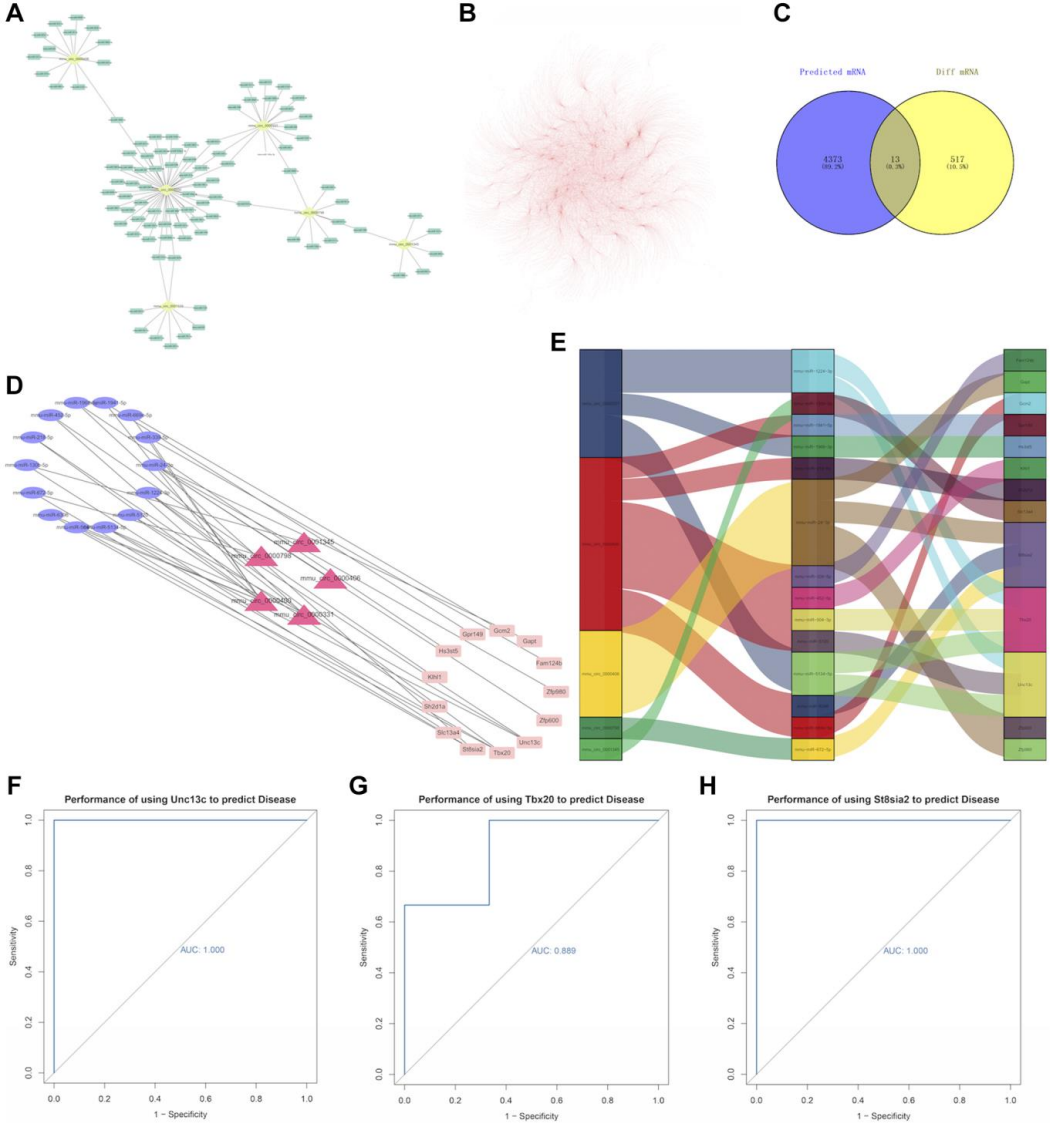
(**A**) Relationship between circRNA and miRNA. The yellow dots represent circRNAs and the green dots represent miRNAs. (**B**) miRNA-mRNA relationship pairs. (**C**) A Venn diagram, in which the purple circle represents 4386 predicted mRNAs, and the yellow circle represents 530 differentially expressed mRNAs in the experiment. Visualization of the ceRNA network. (**D**) The red triangle in the middle represents circRNA, the blue ellipse on the left represents miRNA, and the pink rectangle on the right represents mRNA. (**E**) Use the ggalluvial package for mapping, the longer the box, the more pairs of action relationships. (**F**) ROC curve for Unc13c, (**G**) ROC curve for Tbx20, and (**H**) ROC curve for St8sia2. The larger the area under the curve, the higher the AUC value and the better the prediction efficiency of disease.

In order to find out the downstream genes of circRNA and to construct the ceRNA network, we first used the online circAtlas database (circatlas.biols.ac.cn) to predict miRNA targets for the 5 differentially up-regulated circRNAs with the largest change. The results showed that there were 96 circRNA-related targeted miRNAs, including 105 circRNA-miRNA pairs (Figure 4A). A total of 4,386 mRNAs and 6,725 pairs of miRNA-mRNAs were predicted (Figure 4B). Besides, miRwalk, miRDB, and miRTarBase databases were further used to predict miRNA-related targeted mRNAs, the list of miRNA-mRNA was shown in Appendix 1. Finally, the intersection of 4386 predicted mRNAs and 530 differentially measured mRNAs was obtained, and a total of 13 mRNAs were detected, a Venn diagram was shown in Figure 4C. Then, 19 ceRNA networks were successfully constructed for circRNA-miRNA-mRNA pairs and visualized by the Cytoscape software (Figure 4D). From the Figure 4E we can see that Unc13c, Tbx20, and St8sia2 are located in the largest yellow, pink, and purple regions respectively, so we selected these three genes with the most miRNA relevance as the core genes (mRNA relationship pairs ≥3). We conducted ROC curve analyses to indicate whether three hub genes could better predict PND. The results showed that AUC values of the three hub genes were 1.000 (Figure 4F), 0.889 (Figure 4G), and 1.000 (Figure 4H), respectively. These three hub genes had high diagnostic potential in differentiating aging mice with PND from healthy control mice.

### Immune cell infiltration analysis

**Figure 5:**
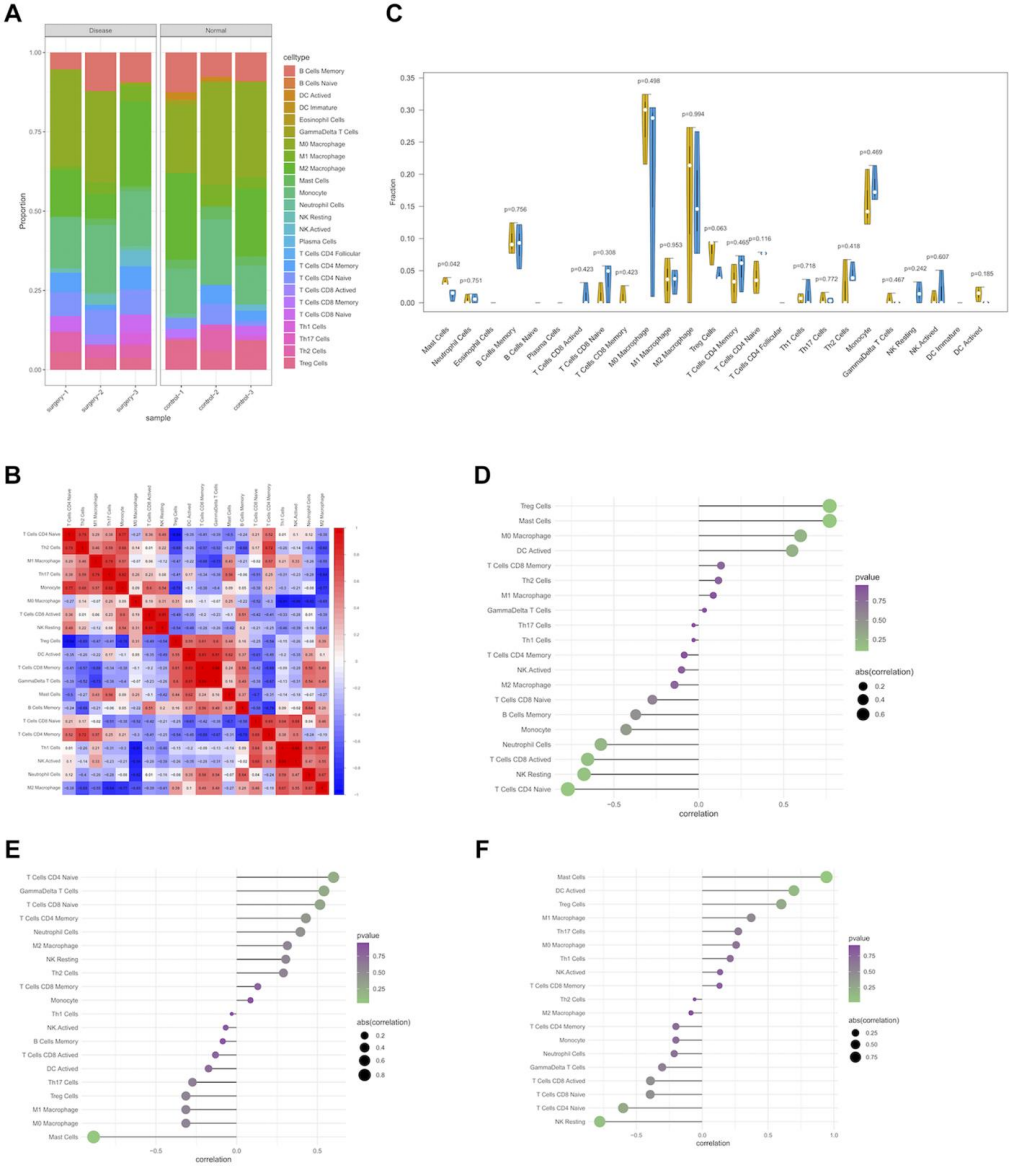
**Immune infiltration of PND group and control group in experimental data.** (**A**) Boxplot of the relative percentage of different types of immune cells in PND and non-PND mice. (**B**) The heat map shows the correlation of CIBERSORT infiltrating innate immune cells, with blue indicating negative correlation and red indicating positive correlation. (**C**) Differences in immune infiltration between PND group (blue) and control group (yellow). (*P* < 0.05 was considered statistically significant). Correlation between expression levels of hub genes and immune cell content using Spearman's correlation. The size of the dot indicates the level of correlation, and the color depth indicates the size of *P*-value. (**D**) The correlation between Unc13C expression and immune cell content (**E**), the correlation between Tbx20 expression and immune cell content (**F**), and the correlation between St8sia2 expression and immune cell content.

Immune microenvironment is mainly composed of immune cells, extracellular matrix, various growth factors, inflammatory factors, and special physical and chemical characteristics, significantly influencing the diagnosis and clinical treatment of diverse diseases. After analyzing the relationship between differentially expressed genes and immune invasion in PND data, the potential molecular mechanism of differentially expressed genes influencing the progression of immune invasion was further discussed. The results of immune cell infiltration assay showed the contents of immune cells in each sample, of which M0 macrophage and M2 macrophage accounted for the highest proportions (Figure 5A), and the correlation between immune cells is shown in Figure 5B. The number of mast cells was significantly lower in patients with disease than that in normal patients (Figure 5C).

We additionally explored the relationship between core genes and immune cells, and the results showed that UNC13C and St8sia2 were significantly positively correlated with Treg cells and mast cells, respectively, and significantly negatively correlated with natural-killer (NK) cells and naive CD4 T cells (Figure 5D, 5F). Tbx20 expression in surgery group significantly increased, it was significantly positively correlated with the number of naive CD4 T cells, and it was significantly negatively correlated with the number of mast cells (Figure 5E). These results confirmed that key genes were closely related to the level of immune cell infiltration and play key roles in the immune microenvironment.

### Correlation analysis of hub genes with chemokines and autophagy-related genes

**Figure 6:**
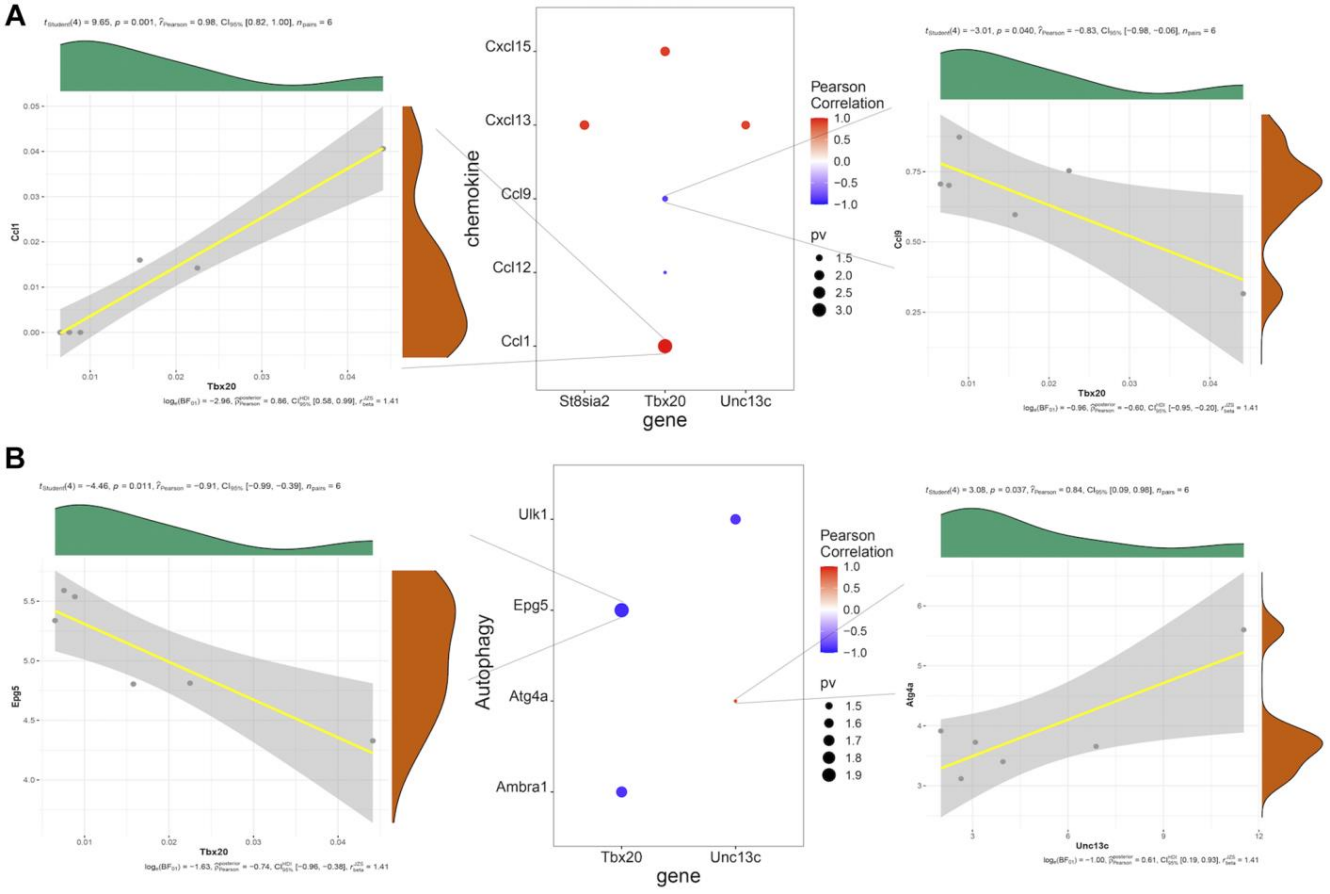
(**A**) Pearson correlation analysis of hub genes and chemokines, in which red represents positive correlation, blue represents negative correlation, and the size of point represents the size of *P*-value. (**B**) Pearson correlation analysis of hub genes and autophagy-related factors, in which red represents positive correlation, blue represents negative correlation, and the size of dot represents the size of *P*-value.

Correlation analysis of hub genes and chemokines showed that Tbx20 expression was significantly positively correlated with Ccl1 expression (Pearson R = 0.98), while Tbx20 expression was significantly negatively correlated with CCL9 expression (Pearson R = −0.83) (Figure 6A). Correlation analysis of hub genes and autophagy-related genes showed that Tbx20 and Epg5 were significantly negatively correlated (Pearson R = −0.91), while Unc13c and Atg4a were significantly positively correlated (Pearson R = 0.84) (Figure 6B).

### GSEA

**Figure 7:**
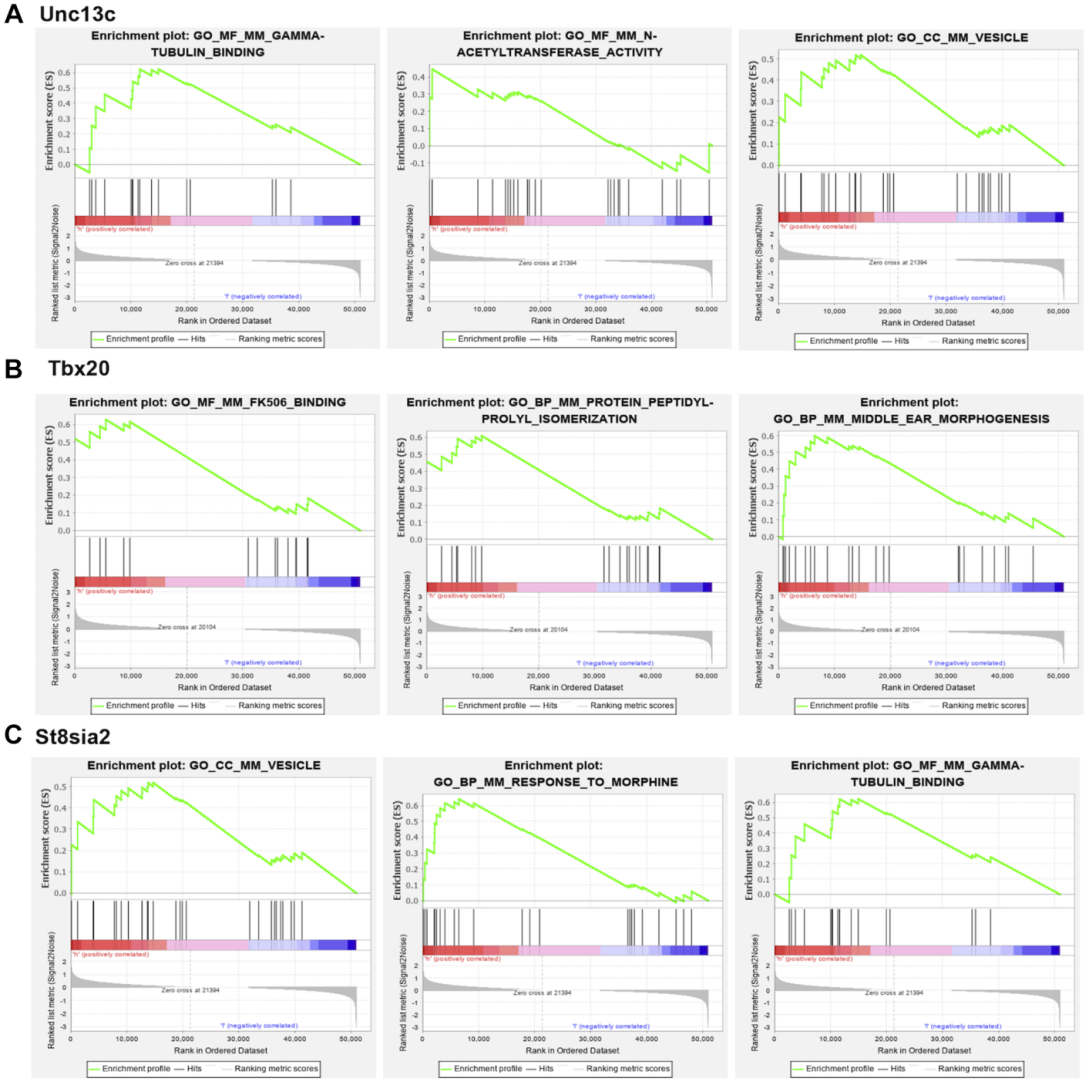
**GSEA analysis of core genes.** (**A**) Unc13c-enriched pathways, including GAMMA-TUBULIN BINDING, N-ACETYL TRANSFERASE ACTIVITY and VESICLE. (**B**) Tbx20-enriched pathways, including FK506 BINDING, PROTEIN PEPTIDYL-PROLYL ISOMERIZATION. (**C**) St8sia2-enriched pathways, including GAMMA-TUBULIN BINDING, VESICLE and RESPONSE TO MORPHINE.

GSEA of the three hub genes showed that high expression of Unc13c was mainly enriched in TUBULIN_BINDING, n-acetyltransferase_activity, VESICLE, and other pathways. The high expression of Tbx20 was mainly concentrated in FK506_BINDING, Protein_Peptidyl-prolyl_isomerization, MIDDLE_EAR_MORPHOGENESIS, and other signaling pathways. The high expression of St8sia2 was mainly concentrated in gamma-tubulin_binding, VESICLR, and response_to_intracestar signaling pathways. Besides, Unc13c, Tbx20, and St8sia2 could regulate the development of PND and affect the course of disease and prognosis of elderly mice through these signaling pathways (Figure 7).

### Identification of the co-expressed genes

**Figure 8:**
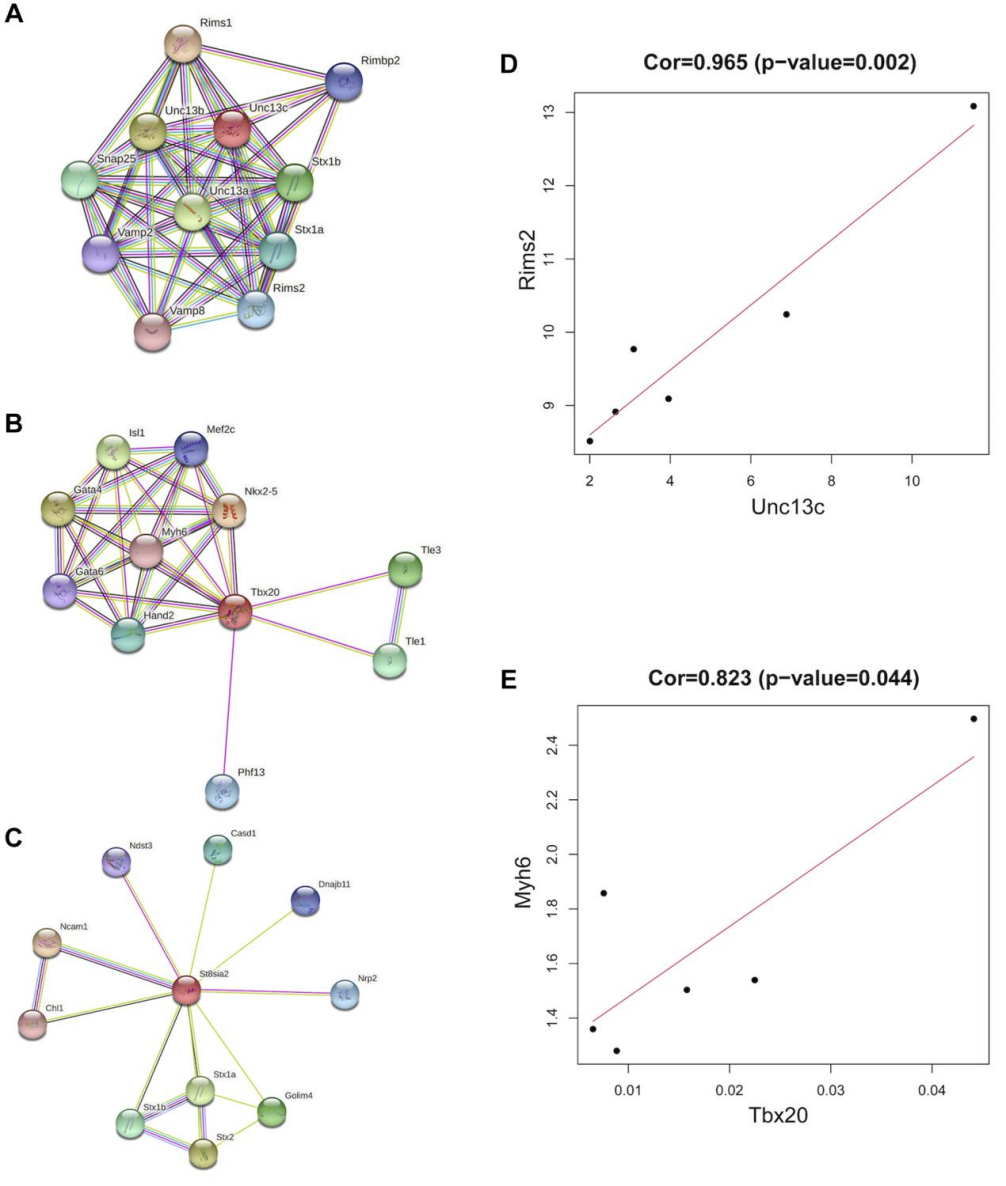
**Co-expression of hub genes.** (**A**) The PPI network plot for Unc13c, including co-expression of 10 predicted genes with Unc13c. (**B**) The PPI network plot for Tbx20, including co-expression of 10 predicted genes with Tbx20. (**C**) The PPI network plot for St8sia2, including co-expression of 10 predicted genes with St8sia2. (**D**) The correlation of Unc13c and Rimsa (**E**) The correlation of Tbx20 and Myh6.

The co-expressed genes (St8sia2, Tbx20, and Unc13c) were retrieved from the String database, and the confidence score was set to 0.4 to establish the corresponding protein interaction network (Figure 8A–8C). In addition, the correlation analysis of the three hub genes and their corresponding co-expressed genes was performed separately, and the results showed that Unc13c and Rims2 were significantly positively correlated, and Tbx20 and Myh6 were significantly negatively correlated. The correlation coefficient between Unc13c and Rims2 was 0.965 (*P* = 0.002) (Figure 8D). The correlation coefficient between Tbx20 and Myh6 was 0.823 (*P* = 0.044) (Figure 8E).

### qRT-PCR and validation

**Figure 9:**
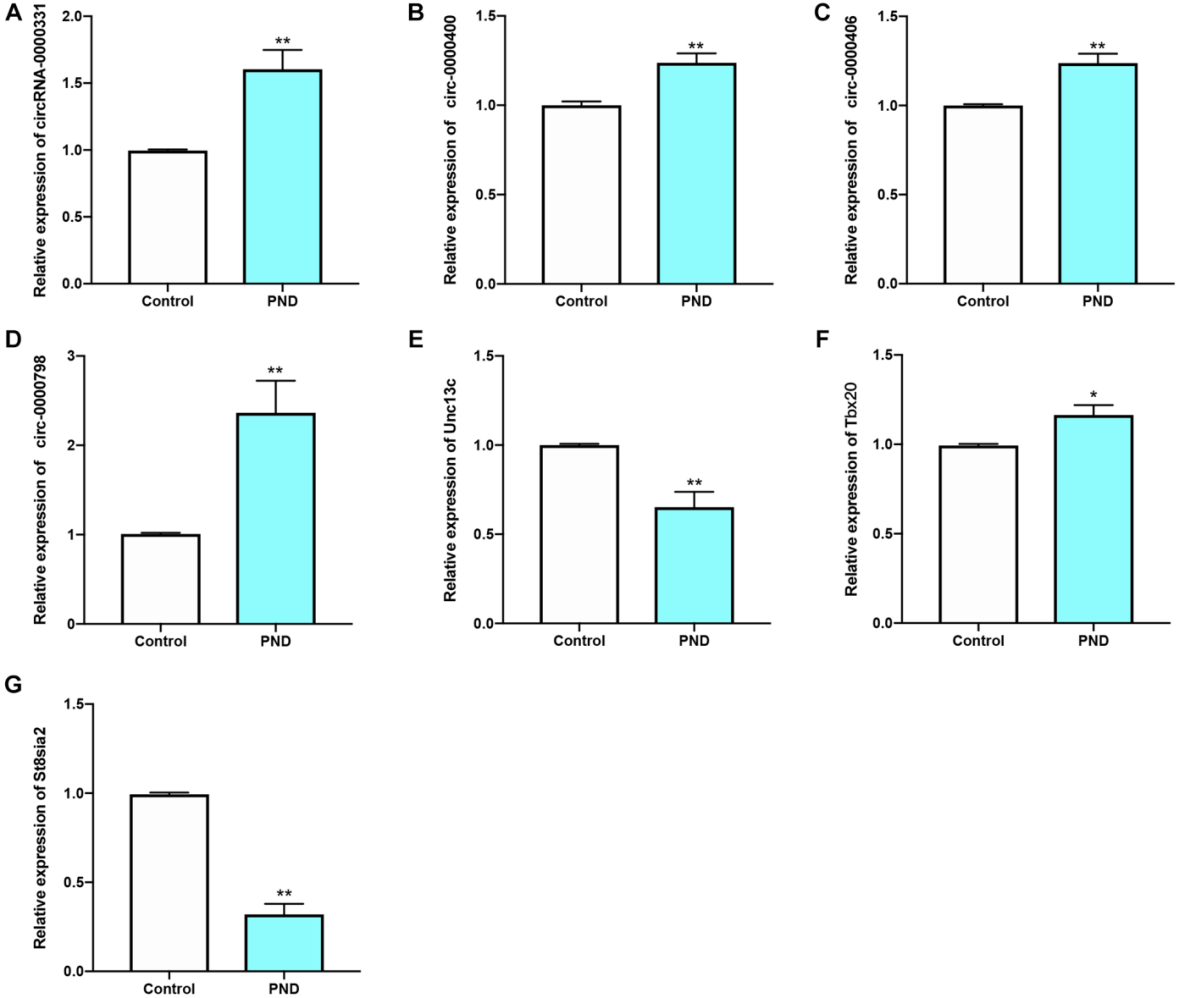
qRT-PCR results of circ-0000331 (**A**); circ-0000400 (**B**); circ-0000406 (**C**); circ-0000798 (**D**); Unc13c (**E**); Tbx20 (**F**); St8sia2 (**G**) were verified in Control and PND groups (*n* = 5/group ). ^*^compared with Control group, *P* < 0.05, ^**^compared with Control group, *P* < 0.01.

The relative expression levels of 4 circRNAs and 3 mRNAs in PND group and control group were detected by qRT-PCR. Compared with the control group, the mRNA expression levels of Unc13c (Figure 9E) and St8sia2 (Figure 9G) in PND group were reduced (*P* < 0.05). The expression levels of CIRC0000331 (Figure 9A), CIRC0000400 (Figure 9B), CIRC0000406 (Figure 9C), CIRC0000798 (Figure 9D), and Tbx20 (Figure 9F) were up-regulated (^#^*P* < 0.05; ^##^*P* < 0.01). These 7 genes could serve as potential biomarkers for diagnosis and prognosis.

### Identification of four bioactive compounds by the CMap analysis

**Figure 10:**
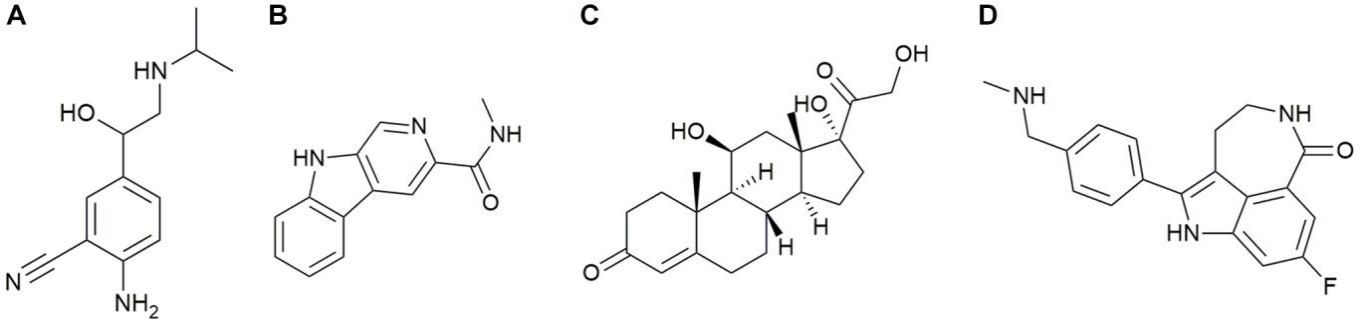
(**A**) Cimaterol (**B**) FG-7142 (**C**) Hydrocortisone (**D**) Rucaparib.

The CMap method was used to select the top 4 active compounds with the largest negative correlation absolute values, including cimaterol, Rucaparib, FG-7142, and Hydrocortisone (Figure 10A–10D), as shown in Appendix 2. These four active compounds could be therapeutic targets of PND.

## DISCUSSION

The course of delirium is acute in PND, mainly appearing 1–3 days after surgery and anesthesia, which is coincident with establishing aging mouse models with PND to screen the maximum severity of PND. However, devastating consequences were obtained, such as the increased mortality in the first year after surgery [19], the decreased quality of life, and the increased long-term risk of Alzheimer’s disease (AD) [20]. In adult mice, circRNA is more abundant in the brain than that in other organs (e.g., heart, liver, and lung) [21]. Cerebral circRNA enrichment is associated with the neurotransmitter function, neuronal maturation, and synaptic activity [22]. CircRNAs have been reported to target aging-related mRNAs in the brain and to regulate the aging process by changing the expressions and availability of specific mRNAs [23]. Based on the important role of circRNAs in regulating the CNS diseases, the current experiment first extracted circRNAs from the GSE174410 dataset, which was combined with our experimental data. Four circRNAs were detected, including mmu_cirC_0000400, mmu_cirC_0000331, mmu_cirC_0000406, and mmu_cirC_0000798, as well as 3 hub genes (Unc13c, Tbx20, and St8sia2).

We first reviewed the literature to understand the three hub genes. Unc13c was widely expressed in 11 tissues, including brain, cerebellum, heart, and liver [24]. Unc13c is a protective gene against AD, and it may be involved in synaptic plasticity and synaptic transmission [25]. This was consistent with the results of the present study, in which Unc13c was down-regulated in the PND group. Unc13c was significantly down-regulated in spinal cord tissues of patients with amyotrophic lateral sclerosis [26]. It was reported as one of the hub genes in a genome-wide association study of post-traumatic stress disorder in Iraq-Afghanistan veterans [27]. Unc13c has also been found to be associated with neurodegeneration in a genetic dementia in Finland [28]. These studies suggested that Unc13c could be closely associated with the CNS, and it could be a target gene for reducing PND. Tbx20 has been detected in unique neurons and epithelial cells of mouse and human embryonic eye tissues [29], associating with the CNS development [30]. Song studied Tbx20 as a gene associated with motor neurons. However, most studies on Tbx20 have concentrated on the cardiovascular system, and Tbx20 was found to play a variety of basic roles in cardiovascular development and homeostasis in response to pathophysiological stress, as well as cardiac remodeling [31, 32]. However, the relationship between Tbx20 and PND needs to be studied by more experiments. St8sia2 syntheses polysialic acid (PSA), which is crucial for cerebral development and is closely related to synaptic plasticity [33]. Lukasz et al. suggested that St8sia2 is a candidate gene that is associated with brain development and plasticity in schizophrenia. Sebastian et al. demonstrated that St8sia2 promotes oligodendrocyte differentiation and myelin repair [34]. Reported in a patient with behavioral disorders, epilepsy and autism spectrum disorders, St8sia2 was expressed in the developing brain, and it showed to play an important role in neuronal migration, axon guidance, and synaptic plasticity [35]. A study on the role of neurodevelopment and genes in psychiatric comorbidity and the regulation of inflammatory process in AD revealed that St8sia2 was only associated with the existence of clinical depression, rather than with AD, and St8sia2 could regulate inflammatory factors (IL-6, IL-1β, etc.) [36]. TANTRA [37] showed that St8sia2 could protect juvenile mice from delayed cognitive impairment several years after cannabis exposure. Taken together, St8sia2 could regulate the cognitive function of the brain, while the specific regulatory mechanism needs more experimental studies. As a result, we found that Unc13c and St8sia2 were closely associated with the CNS and cognitive function, and they could regulate PND.

The results of GO and KEGG pathway enrichment analyses revealed that, 530 differentially expressed genes were enriched in B cell proliferation and transmembrane receptor protein serine/threonine kinase signaling pathway functions. This suggested that PND could be related to immunity. Few studies have systematically screened biomarkers associated with PND immune infiltration. To further explore the role of immune cell infiltration in PND, CIBERSORT was used to comprehensively evaluate PND. The results showed that the number of mast cells in PND group was significantly reduced compared with that in the normal group. This is consistent with the results of a study on depression: isolated social stress caused depression in mice, significantly reducing the total number of mast cells in the brain by 90% on the first day [38]. There are also conflicting and controversial findings about the role of mast cells.

On the one hand, mast cells seem to mediate neuroinflammation protection. Mast cells release specific lyase McP-4 to degrade inflammatory cytokines in a mouse model of traumatic brain injury (TBI) to modulate the CNS disease [39]. Some proteases released by mast cells play homeostasis, protection, and even anti-inflammatory roles [40]. On the other hand, palmitoethanolamide (PEA) induced attenuation of the number of mast cells, amylase and trypsin, and increased cerebral edema, infarct volume, and brain injury in experimental TBI mice [41]. Mast cells release mediators into the CNS, promoting neurogenesis, such as serotonin and IL-6, providing neuroprotection (e.g., IL-1β), and maintaining the integrity of the blood-brain barrier (BBB), such as histamine. However, excessive levels of these mediators have detrimental effects on the integrity of neurons and the BBB. These results seem to be contradictory, whereas the effect of inflammation on TBI may be beneficial or detrimental depending on the time after injury and the stage of TBI. Logically speaking, the role of mast cells in these different stages is two-fold [42]. The results of the correlation between Unc13c and St8sia2 and immune cells showed that the combination of Unc13c and St8sia2 significantly decreased in the surgery group compared with that in the control group, suggesting that Unc13c and St8sia2 could protect PND by regulating immunity. In recent years, the role of T cells in neurodegenerative diseases has noticeably attracted scholars’ attention [15]. In healthy cerebrospinal fluid without inflammation, 90% of cells were reported to be T cells, mainly CD4 cells [43]. In pathological conditions, T cells can penetrate the brain parenchyma, while CD4+ T cells may play a role in the pro-inflammatory process of postoperative cognitive dysfunction [44]. Regulatory CD4 cells (Treg cells) provide neuroprotection by attenuating microglial activation in the CNS diseases [45]. Zhu et al. [44] demonstrated that PND is related to the increased number of T cells and NK cells in the hippocampus of aging mice after surgery and anesthesia. NK cell involvement in neurodegenerative diseases, such as multiple sclerosis and AD, has been reported [46]. The reduction of NK cell activity was previously found in AD patients [47]. It is noteworthy that NK cell infiltration into the BBB may not be a defense response, while it could be the result of PND progression, leading to immune system activation. The results of qRT-PCR and Western blotting of Tbx20 combined with the correlation of Tbx20 immune cells suggested that Tbx20 might be a hub gene, promoting PND. Chemokines are a large family of small cytokines that control the migration and residence of all immune cells. The CXC family includes chemokines CXCL1–CXCL17, and the CC family includes CCL1-CCL28. CC chemokines not only stimulate monocytes, but also basophils, eosinophils, T lymphocytes, and NK cells [48]. Studies have shown that changes in chemokines and chemokine receptors play an important role in the inflammatory response to AD, in which CCL1 and CCR8 prevent bacterial infection from participating in the demyelination of AD. The results of the present study showed that Tbx20 was positively correlated with CCL1, suggesting that Tbx20 could regulate immunity and participate in the development of neurodegenerative diseases. Unc13c and St8sia2 were significantly correlated with CCL9, which could be closely related to PND [49, 50]. These evidences suggested that the three hub genes, which are closely related to levels of immune cell infiltration, play a critical role in the immune microenvironment. It is possible to influence the occurrence of PND by regulating the whole immune process.

Autophagy is an important pathway to eliminate abnormal protein aggregation in mammalian cells, which is related to protein homeostasis and neuronal health. Several studies have recently shown a close relationship between autophagy and PND [10, 51, 52]. Based on the close relationship between the transmembrane receptor protein serine/threonine kinase pathway of GO function and mTOR and autophagy, we conducted correlation analysis of hub genes and autophagy-related genes, and found that Unc13c, Tbx20, and St8sia2 were significantly correlated with different autophagy-related factors. Therefore, we speculated that these hub genes might regulate PND in aging mice through autophagy.

Finally, nine groups of ceRNA networks were identified, including mmu_circ_0000331/miR-1224-3p/Unc13c, mmu_circ_0000400/miR-5120/Unc13c, mmu_circ_0000331/miR-5134-5p/Unc13c, mmu_circ_0000331/miR-1224-3p/Tbx20, mmu_circ_0000400/miR-504-3p/Tbx20; mmu_circ_0000331/miR-5134-5p/Tbx20, mmu_circ_0000406/miR-24-3p/St8sia2, mmu_circ_0000400/miR-6396/St8sia2, and mmu_circ_0000798/miR-672-5p/St8sia2. Through literature review of each circRNA and miRNA, miR-24-3p was found to be closely related to AD [53–55]. Among them, miR-24-3p in Liu et al.’s study was noted to be negatively correlated with the score of mild mental state testing of AD, and down-regulation of miR-24-3p could promote cell proliferation and inhibit cell apoptosis [56]. Zhang et al.’s findings were different, in which mice with AD showed a decreasing trend of miR-24-3p with age [57]. These results suggest that miR-24-3p may be closely related to PND. In addition, miR-1224-3p was found to be highly expressed in the hippocampus, and the decrease of miR-1224-3p could promote the growth and metastasis of glioma cells [58, 59]. Other circRNAs and miRNAs have not been found in studies on the CNS diseases. Hence, we will concentrate on the regulatory roles of mmu_circ_0000331/miR-1224-3p/Unc13c and mmu_circ_0000406/mmu-miR-24-3p/St8sia2 in PND.

### Limitations

As of the time of our analysis of this project, only one circRNA dataset (GSE174410) on postoperative neurocognitive disorder was available in the GEO public database. Therefore, subsequent studies by our group will also be conducted based on this dataset. If more circRNA datasets can be selected in the GEO database, the bias of the experiment can be reduced. In addition, the four drugs we screened will be validated in the follow-up study; and the mechanism of action of screening PND-related ceRNAs will also be studied in more depth.

## CONCLUSIONS

In summary, we detected three mRNAs and four circRNAs that were associated with immune regulation of PND using publicly available data and combined mRNA analysis of high-throughput sequencing of aging mouse hippocampus after splenectomy by our experimental data. The results of the qRT-PCR verified the differential expression of these seven genes in the control and surgical groups, and Western blotting re-verified the differential expression of the three mRNAs in the two groups. After reviewing the literature to remove the genes that were not associated with the CNS, two new ceRNA hub regulatory networks were finally constructed. The data of differentially expressed genes measured by our experiments were analyzed using CMap, and four active compounds (cimaterol, rucaparib, FG-7142, and hydrocortisone) were detected to interfere with gene expression. The results of the the present study may provide new insights into the immunomodulation-related pathogenesis and potential therapeutic targets of PND.

## References

[1] Berger M, Schenning KJ, Brown CH 4th, Deiner SG, Whittington RA, Eckenhoff RG, Angst MS, Avramescu S, Bekker A, Brzezinski M, Crosby G, Culley DJ, Eckenhoff M, et al, and Perioperative Neurotoxicity Working Group. Best Practices for Postoperative Brain Health: Recommendations From the Fifth International Perioperative Neurotoxicity Working Group. Anesth Analg. 2018; 127:1406–13. 10.1213/ANE.0000000000003841 30303868 PMC6309612

[2] Subramaniyan S, Terrando N. Neuroinflammation and Perioperative Neurocognitive Disorders. Anesth Analg. 2019; 128:781–8. 10.1213/ANE.0000000000004053 30883423 PMC6437083

[3] Choi S, Jerath A, Jones P, Avramescu S, Djaiani G, Syed S, Saha T, Kaustov L, Kiss A, D'Aragon F, Hedlin P, Rajamohan R, Couture EJ, et al. Cognitive Outcomes after DEXmedetomidine sedation in cardiac surgery: CODEX randomised controlled trial protocol. BMJ Open. 2021; 11:e046851. 10.1136/bmjopen-2020-046851 33849856 PMC8051371

[4] Leslie M. The post-op brain. Science. 2017; 356:898–900. 10.1126/science.356.6341.898 28572347

[5] Wei P, Yang F, Zheng Q, Tang W, Li J. The Potential Role of the NLRP3 Inflammasome Activation as a Link Between Mitochondria ROS Generation and Neuroinflammation in Postoperative Cognitive Dysfunction. Front Cell Neurosci. 2019; 13:73. 10.3389/fncel.2019.00073 30873011 PMC6401615

[6] Belrose JC, Noppens RR. Anesthesiology and cognitive impairment: a narrative review of current clinical literature. BMC Anesthesiol. 2019; 19:241. 10.1186/s12871-019-0903-7 31881996 PMC6933922

[7] Zeng W, Zhang C, Long Q, Li Y. Dexmedetomidine Alleviates LPS-Induced Neuronal Dysfunction by Modulating the AKT/GSK-3β/CRMP-2 Pathway in Hippocampal Neurons. Neuropsychiatr Dis Treat. 2021; 17:671–80. 10.2147/NDT.S297365 33727816 PMC7955869

[8] Li J, Shi C, Ding Z, Jin W. Glycogen Synthase Kinase 3*β* Promotes Postoperative Cognitive Dysfunction by Inducing the M1 Polarization and Migration of Microglia. Mediators Inflamm. 2020; 2020:7860829. 10.1155/2020/7860829 33354162 PMC7735842

[9] Wang YL, Zhang Y, Cai DS. Dexmedetomidine Ameliorates Postoperative Cognitive Dysfunction via the MicroRNA-381-Mediated EGR1/p53 Axis. Mol Neurobiol. 2021; 58:5052–66. 10.1007/s12035-021-02417-7 34245441

[10] Lan N, Liu Y, Juan Z, Zhang R, Ma B, Xie K, Sun L, Feng H, Sun M, Liu J. The TSPO-specific Ligand PK11195 Protects Against LPS-Induced Cognitive Dysfunction by Inhibiting Cellular Autophagy. Front Pharmacol. 2021; 11:615543. 10.3389/fphar.2020.615543 33708121 PMC7941270

[11] Chen SM, Li M, Xie J, Li S, Xiang SS, Liu HY, Chen Z, Zhang P, Kuang X, Tang XQ. Hydrogen sulfide attenuates postoperative cognitive dysfunction through promoting the pathway of Warburg effect-synaptic plasticity in hippocampus. Toxicol Appl Pharmacol. 2020; 409:115286. 10.1016/j.taap.2020.115286 33068621

[12] Ma N, Tie C, Yu B, Zhang W, Wan J. Identifying lncRNA-miRNA-mRNA networks to investigate Alzheimer's disease pathogenesis and therapy strategy. Aging (Albany NY). 2020; 12:2897–20. 10.18632/aging.102785 32035423 PMC7041741

[13] Wu YQ, Liu Q, Wang HB, Chen C, Huang H, Sun YM, Ma LH, Wan J, Sun YY, Miao HH. Microarray Analysis Identifies Key Differentially Expressed Circular RNAs in Aged Mice With Postoperative Cognitive Dysfunction. Front Aging Neurosci. 2021; 13:716383. 10.3389/fnagi.2021.716383 34483886 PMC8415796

[14] Umholtz M, Nader ND. Anesthetic Immunomodulation of the Neuroinflammation in Postoperative Cognitive Dysfunction. Immunol Invest. 2017; 46:805–15. 10.1080/08820139.2017.1373898 29058541

[15] Liu Y, Yin Y. Emerging Roles of Immune Cells in Postoperative Cognitive Dysfunction. Mediators Inflamm. 2018; 2018:6215350. 10.1155/2018/6215350 29670465 PMC5835271

[16] Cibelli M, Fidalgo AR, Terrando N, Ma D, Monaco C, Feldmann M, Takata M, Lever IJ, Nanchahal J, Fanselow MS, Maze M. Role of interleukin-1beta in postoperative cognitive dysfunction. Ann Neurol. 2010; 68:360–8. 10.1002/ana.22082 20818791 PMC4836445

[17] Kamer AR, Galoyan SM, Haile M, Kline R, Boutajangout A, Li YS, Bekker A. Meloxicam improves object recognition memory and modulates glial activation after splenectomy in mice. Eur J Anaesthesiol. 2012; 29:332–7. 10.1097/EJA.0b013e3283534f56 22513481

[18] Lamb J, Crawford ED, Peck D, Modell JW, Blat IC, Wrobel MJ, Lerner J, Brunet JP, Subramanian A, Ross KN, Reich M, Hieronymus H, Wei G, et al. The Connectivity Map: using gene-expression signatures to connect small molecules, genes, and disease. Science. 2006; 313:1929–35. 10.1126/science.1132939 17008526

[19] Koster S, Hensens AG, Schuurmans MJ, van der Palen J. Consequences of delirium after cardiac operations. Ann Thorac Surg. 2012; 93:705–11. 10.1016/j.athoracsur.2011.07.006 21992939

[20] Inouye SK, Marcantonio ER, Kosar CM, Tommet D, Schmitt EM, Travison TG, Saczynski JS, Ngo LH, Alsop DC, Jones RN. The short-term and long-term relationship between delirium and cognitive trajectory in older surgical patients. Alzheimers Dement. 2016; 12:766–75. 10.1016/j.jalz.2016.03.005 27103261 PMC4947419

[21] You X, Vlatkovic I, Babic A, Will T, Epstein I, Tushev G, Akbalik G, Wang M, Glock C, Quedenau C, Wang X, Hou J, Liu H, et al. Neural circular RNAs are derived from synaptic genes and regulated by development and plasticity. Nat Neurosci. 2015; 18:603–10. 10.1038/nn.3975 25714049 PMC4376664

[22] Mahmoudi E, Fitzsimmons C, Geaghan MP, Shannon Weickert C, Atkins JR, Wang X, Cairns MJ. Circular RNA biogenesis is decreased in postmortem cortical gray matter in schizophrenia and may alter the bioavailability of associated miRNA. Neuropsychopharmacology. 2019; 44:1043–54. 10.1038/s41386-019-0348-1 30786269 PMC6461776

[23] Mahmoudi E, Cairns MJ. Circular RNAs are temporospatially regulated throughout development and ageing in the rat. Sci Rep. 2019; 9:2564. 10.1038/s41598-019-38860-9 30796328 PMC6385508

[24] Zhong YJ, Yang Y, Wang XY, Di R, Chu MX, Liu QY. Expression analysis and single-nucleotide polymorphisms of *SYNDIG1L* and *UNC13C* genes associated with thoracic vertebral numbers in sheep (*Ovis aries*). Arch Anim Breed. 2021; 64:131–8. 10.5194/aab-64-131-2021 34084911 PMC8131962

[25] Miller JA, Woltjer RL, Goodenbour JM, Horvath S, Geschwind DH. Genes and pathways underlying regional and cell type changes in Alzheimer's disease. Genome Med. 2013; 5:48. 10.1186/gm452 23705665 PMC3706780

[26] D'Erchia AM, Gallo A, Manzari C, Raho S, Horner DS, Chiara M, Valletti A, Aiello I, Mastropasqua F, Ciaccia L, Locatelli F, Pisani F, Nicchia GP, et al. Massive transcriptome sequencing of human spinal cord tissues provides new insights into motor neuron degeneration in ALS. Sci Rep. 2017; 7:10046. 10.1038/s41598-017-10488-7 28855684 PMC5577269

[27] Ashley-Koch AE, Garrett ME, Gibson J, Liu Y, Dennis MF, Kimbrel NA, Beckham JC, Hauser MA, and Veterans Affairs Mid-Atlantic Mental Illness Research, Education, and Clinical Center Workgroup. Genome-wide association study of posttraumatic stress disorder in a cohort of Iraq-Afghanistan era veterans. J Affect Disord. 2015; 184:225–34. 10.1016/j.jad.2015.03.049 26114229 PMC4697755

[28] Pasanen P, Myllykangas L, Pöyhönen M, Kiviharju A, Siitonen M, Hardy J, Bras J, Paetau A, Tienari PJ, Guerreiro R, Verkkoniemi-Ahola A. Genetics of dementia in a Finnish cohort. Eur J Hum Genet. 2018; 26:827–37. 10.1038/s41431-018-0117-3 29476165 PMC5974394

[29] Das S, Chen QB, Saucier JD, Drescher B, Zong Y, Morgan S, Forstall J, Meriwether A, Toranzo R, Leal SM. The Drosophila T-box transcription factor Midline functions within the Notch-Delta signaling pathway to specify sensory organ precursor cell fates and regulates cell survival within the eye imaginal disc. Mech Dev. 2013; 130:577–601. 10.1016/j.mod.2013.08.001 23962751 PMC4500660

[30] Yu J, Mu J, Guo Q, Yang L, Zhang J, Liu Z, Yu B, Zhang T, Xie J. Transcriptomic profile analysis of mouse neural tube development by RNA-Seq. IUBMB Life. 2017; 69:706–19. 10.1002/iub.1653 28691208

[31] Chen Y, Xiao D, Zhang L, Cai CL, Li BY, Liu Y. The Role of *Tbx20* in Cardiovascular Development and Function. Front Cell Dev Biol. 2021; 9:638542. 10.3389/fcell.2021.638542 33585493 PMC7876368

[32] Luyckx I, Kumar AA, Reyniers E, Dekeyser E, Vanderstraeten K, Vandeweyer G, Wünnemann F, Preuss C, Mazzella JM, Goudot G, Messas E, Albuisson J, Jeunemaitre X, et al, and MIBAVA Leducq Consortium. Copy number variation analysis in bicuspid aortic valve-related aortopathy identifies TBX20 as a contributing gene. Eur J Hum Genet. 2019; 27:1033–43. 10.1038/s41431-019-0364-y 30820038 PMC6777542

[33] Ikegami K, Saigoh K, Fujioka A, Nagano M, Kitajima K, Sato C, Masubuchi S, Kusunoki S, Shigeyoshi Y. Effect of expression alteration in flanking genes on phenotypes of St8sia2-deficient mice. Sci Rep. 2019; 9:13634. 10.1038/s41598-019-50006-5 31541165 PMC6754417

[34] Werneburg S, Fuchs HLS, Albers I, Burkhardt H, Gudi V, Skripuletz T, Stangel M, Gerardy-Schahn R, Hildebrandt H. Polysialylation at Early Stages of Oligodendrocyte Differentiation Promotes Myelin Repair. J Neurosci. 2017; 37:8131–41. 10.1523/JNEUROSCI.1147-17.2017 28760868 PMC6596786

[35] Kamien B, Harraway J, Lundie B, Smallhorne L, Gibbs V, Heath A, Fullerton JM. Characterization of a 520 kb deletion on chromosome 15q26.1 including ST8SIA2 in a patient with behavioral disturbance, autism spectrum disorder, and epilepsy. Am J Med Genet A. 2014; 164A:782–8. 10.1002/ajmg.a.36345 24357335

[36] Porcelli S, Crisafulli C, Donato L, Calabrò M, Politis A, Liappas I, Albani D, Atti AR, Salfi R, Raimondi I, Forloni G, Papadimitriou GN, De Ronchi D, Serretti A. Role of neurodevelopment involved genes in psychiatric comorbidities and modulation of inflammatory processes in Alzheimer's disease. J Neurol Sci. 2016; 370:162–6. 10.1016/j.jns.2016.09.053 27772752

[37] Tantra M, Kröcher T, Papiol S, Winkler D, Röckle I, Jatho J, Burkhardt H, Ronnenberg A, Gerardy-Schahn R, Ehrenreich H, Hildebrandt H. St8sia2 deficiency plus juvenile cannabis exposure in mice synergistically affect higher cognition in adulthood. Behav Brain Res. 2014; 275:166–75. 10.1016/j.bbr.2014.08.062 25200516

[38] Bugajski AJ, Chłap Z, Gadek-Michalska, Bugajski J. Effect of isolation stress on brain mast cells and brain histamine levels in rats. Agents Actions. 1994; 41:C75–6. 10.1007/BF02007774 7526664

[39] Stokely ME, Orr EL. Acute effects of calvarial damage on dural mast cells, pial vascular permeability, and cerebral cortical histamine levels in rats and mice. J Neurotrauma. 2008; 25:52–61. 10.1089/neu.2007.0397 18355158

[40] Caughey GH. Mast cell proteases as pharmacological targets. Eur J Pharmacol. 2016; 778:44–55. 10.1016/j.ejphar.2015.04.045 25958181 PMC4636979

[41] Ahmad A, Crupi R, Impellizzeri D, Campolo M, Marino A, Esposito E, Cuzzocrea S. Administration of palmitoylethanolamide (PEA) protects the neurovascular unit and reduces secondary injury after traumatic brain injury in mice. Brain Behav Immun. 2012; 26:1310–21. 10.1016/j.bbi.2012.07.021 22884901

[42] Hendriksen E, van Bergeijk D, Oosting RS, Redegeld FA. Mast cells in neuroinflammation and brain disorders. Neurosci Biobehav Rev. 2017; 79:119–33. 10.1016/j.neubiorev.2017.05.001 28499503

[43] Kivisäkk P, Mahad DJ, Callahan MK, Trebst C, Tucky B, Wei T, Wu L, Baekkevold ES, Lassmann H, Staugaitis SM, Campbell JJ, Ransohoff RM. Human cerebrospinal fluid central memory CD4+ T cells: evidence for trafficking through choroid plexus and meninges via P-selectin. Proc Natl Acad Sci U S A. 2003; 100:8389–94. 10.1073/pnas.1433000100 12829791 PMC166239

[44] Zhu H, Liu W, Fang H. Inflammation caused by peripheral immune cells across into injured mouse blood brain barrier can worsen postoperative cognitive dysfunction induced by isoflurane. BMC Cell Biol. 2018; 19:23. 10.1186/s12860-018-0172-1 30268095 PMC6162931

[45] Reynolds AD, Stone DK, Hutter JA, Benner EJ, Mosley RL, Gendelman HE. Regulatory T cells attenuate Th17 cell-mediated nigrostriatal dopaminergic neurodegeneration in a model of Parkinson's disease. J Immunol. 2010; 184:2261–71. 10.4049/jimmunol.0901852 20118279 PMC2824790

[46] Trachtenberg EA. Understanding the role of natural killer cell receptors and their human leukocyte antigen ligands in multiple sclerosis. Ann Neurol. 2009; 65:626–8. 10.1002/ana.21747 19557875

[47] Richartz-Salzburger E, Batra A, Stransky E, Laske C, Köhler N, Bartels M, Buchkremer G, Schott K. Altered lymphocyte distribution in Alzheimer's disease. J Psychiatr Res. 2007; 41:174–8. 10.1016/j.jpsychires.2006.01.010 16516234

[48] Palomino DC, Marti LC. Chemokines and immunity. Einstein (Sao Paulo). 2015; 13:469–73. 10.1590/S1679-45082015RB3438 26466066 PMC4943798

[49] Shen Y, Zhang Y, Chen L, Du J, Bao H, Xing Y, Cai M, Si Y. Chemokine CXCL13 acts via CXCR5-ERK signaling in hippocampus to induce perioperative neurocognitive disorders in surgically treated mice. J Neuroinflammation. 2020; 17:335. 10.1186/s12974-020-02013-x 33161894 PMC7648984

[50] Xie HH, Ma HY, Zhang S, Li JW, Han Q, Chen HQ, Su BQ, Zhou JP. Impact of edaravone on serum CXC chemokine ligand-13 levels and perioperative neurocognitive disorders in elderly patients with hip replacement. Chin Med J (Engl). 2021; 134:1610–5. 10.1097/CM9.0000000000001492 34133348 PMC8280092

[51] Gao S, Zhang S, Zhou H, Tao X, Ni Y, Pei D, Kang S, Yan W, Lu J. Role of mTOR-Regulated Autophagy in Synaptic Plasticity Related Proteins Downregulation and the Reference Memory Deficits Induced by Anesthesia/Surgery in Aged Mice. Front Aging Neurosci. 2021; 13:628541. 10.3389/fnagi.2021.628541 33935683 PMC8085306

[52] Yang N, Li Z, Han D, Mi X, Tian M, Liu T, Li Y, He J, Kuang C, Cao Y, Li L, Ni C, Wang JQ, Guo X. Autophagy prevents hippocampal α-synuclein oligomerization and early cognitive dysfunction after anesthesia/surgery in aged rats. Aging (Albany NY). 2020; 12:7262–81. 10.18632/aging.103074 32335546 PMC7202547

[53] Lu L, Dai WZ, Zhu XC, Ma T. Analysis of Serum miRNAs in Alzheimer's Disease. Am J Alzheimers Dis Other Demen. 2021; 36:1–9. 10.1177/15333175211021712 34080437 PMC10581118

[54] Ansari A, Maffioletti E, Milanesi E, Marizzoni M, Frisoni GB, Blin O, Richardson JC, Bordet R, Forloni G, Gennarelli M, Bocchio-Chiavetto L, and PharmaCog Consortium. miR-146a and miR-181a are involved in the progression of mild cognitive impairment to Alzheimer's disease. Neurobiol Aging. 2019; 82:102–9. 10.1016/j.neurobiolaging.2019.06.005 31437718

[55] Rahman MR, Islam T, Zaman T, Shahjaman M, Karim MR, Huq F, Quinn JMW, Holsinger RMD, Gov E, Moni MA. Identification of molecular signatures and pathways to identify novel therapeutic targets in Alzheimer's disease: Insights from a systems biomedicine perspective. Genomics. 2020; 112:1290–9. 10.1016/j.ygeno.2019.07.018 31377428

[56] Liu L, Liu L, Lu Y, Zhang T, Zhao W. Serum aberrant expression of miR-24-3p and its diagnostic value in Alzheimer's disease. Biomark Med. 2021; 15:1499–507. 10.2217/bmm-2021-0098 34668391

[57] Zhang T, Shen Y, Guo Y, Yao J. Identification of key transcriptome biomarkers based on a vital gene module associated with pathological changes in Alzheimer's disease. Aging (Albany NY). 2021; 13:14940–67. 10.18632/aging.203017 34031265 PMC8221319

[58] Du S, Li H, Lu F, Zhang S, Tang J. Circular RNA ZNF609 promotes the malignant progression of glioma by regulating miR-1224-3p/PLK1 signaling. J Cancer. 2021; 12:3354–66. 10.7150/jca.54934 33976745 PMC8100806

[59] Wang Y, Wang M, Wei W, Han D, Chen X, Hu Q, Yu T, Liu N, You Y, Zhang J. Disruption of the EZH2/miRNA/β-catenin signaling suppresses aerobic glycolysis in glioma. Oncotarget. 2016; 7:49450–8. 10.18632/oncotarget.10370 27385092 PMC5226520

